# Supplementary material for: Exploring the relationship between collaterals and vessel density in retinal vein occlusions using optical coherence tomography angiography
Source: PLoS One. 2019 Jul 24;14(7):e0215790. doi: 10.1371/journal.pone.0215790 (PMC6655539; doi:10.1371/journal.pone.0215790)
Supplement: S1 Table — (DOCX) [file pone.0215790.s001.docx]

| **S1 Table. Results of Poisson Regression Analysis to Study the Association between Vessel Density and Number of Collaterals in Eyes with Retinal Vein Occlusion** | | | | | |
| --- | --- | --- | --- | --- | --- |
| **Covariates** | **Estimated regression coefficient (β)** | **Χ^2^** | **P-value*** | **Estimated IRR** | **95% CI for IRR** |
| SCP VD | -0.152 | 11.94 | < 0.001 | 0.86 | 0.79 – 0.94 |
| DCP VD | 0.004 | 0.02 | 0.899 | 1.00 | 0.94 – 1.08 |
| SCP= superficial capillary plexus; DCP= deep capillary plexus; VD= parafoveal vessel density; IRR= incidence rate ratio obtained by exponentiating the Poisson regression coefficient (β); Χ2= Wald chi-square; CI= Wald confidence interval  P-value < 0.05 was considered statistically significant.  * GEE correction for clustering of data into RVO types (BRVO, CRVO, HRVO). | | | | | |
